# Effect of vitamin D fortified foods on bone markers and muscle strength in women of Pakistani and Danish origin living in Denmark: a randomised controlled trial

**DOI:** 10.1186/s12937-019-0504-9

**Published:** 2019-12-02

**Authors:** Ida M. Grønborg, Inge Tetens, Elisabeth Wreford Andersen, Michael Kristensen, Rikke E. K. Larsen, Thanh L. L. Tran, Rikke Andersen

**Affiliations:** 1 National Food Institute, Technical University of Denmark, Lyngby, Denmark; 2 Division Diet, Disease Prevention and Toxicology, National Food Institute, Technical University of Denmark, Lyngby, Denmark; 3 Vitality - Centre for good older lives, Department of Nutrition, Exercise and Sports, University of Copenhagen, Frederiksberg C, Denmark; 4 Section for Statistics and Pharmacoepidemiology, Danish Cancer Society, Copenhagen, Denmark; 5 Institute of Applied Mathematics and Computer Science, Technical University of Denmark, Lyngby, Denmark; 6 Department of Nursing and Nutrition, Faculty of Health, University College Copenhagen, Copenhagen, Denmark

**Keywords:** Vitamin D, Ranomised controlled trial, Immigrants, Food, Fortified, Muscle strength, Bone resorption, Bone regeneration

## Abstract

**Background:**

Deficient and insufficient vitamin D status (defined as serum 25(OH)D < 30 nmol/L and > 50 nmol/L) is prevalent worldwide and associated with decreased muscle strength and poor bone health. We aimed to investigate the effect of vitamin D fortification on bone markers and muscle strength among younger adult women at risk of vitamin D deficiency.

**Methods:**

A 12-week randomised double-blinded placebo-controlled winter intervention trial, providing 30 μg vitamin D_3_/day through fortified yoghurt, cheese, eggs and crisp-bread or similar placebo products. Participants were 143 women of Danish and Pakistani origin 18–50 years of age, living in Denmark, randomised into four groups stratified by ethnicity. Serum 25-hydroxyvitamin D (25(OH)D) by LC-MS/MS and the secondary endpoints: four specific bone markers (osteocalcin (OC), Bone specific Alkaline Phosphatase (BALP), Procollagen type 1 amino-terminal propeptide (P1NP), C-terminal crosslinked telopeptide of type 1 collagen (CTX)) and three muscle strength measures (handgrip, knee extension strength, chair-standing), were assessed using one-way ANOVA, Tukey HSD and subsequent linear ANCOVA models, adjusted for relevant covariates.

**Results:**

Significantly increased serum 25(OH)D concentration from 53.3 (17) to 77.8 (14) nmol/L and from 44.5 (21) to 54.7 (18) nmol/L among Danish and Pakistani women in the fortified groups, respectively (*P* <  0.05). The bone turnover markers OC, BALP, P1NP and CTX did not change significantly. Muscle strength by handgrip, knee extension and chair-standing test did not change significantly following the intervention.

**Conclusions:**

Consumption of vitamin D fortified foods for 12 weeks did not result in significant changes of the bone turnover markers OC, BALP, P1NP and CTX. Muscle strength measured as hand grip strength, knee extension strength and chair-standing did not change significantly following the intervention.

## Introduction

Vitamin D has a well-established role in the calcium and phosphorus homeostasis and is essential for bone health [1, 2]. Studies have shown associations between vitamin D deficiency and insufficiency, here and throughout this paper, defined as serum 25(OH)D concentrations < 30 and < 50 nmol/L, respectively [3, 4], and non-skeletal endpoints such as muscular atrophy, muscle pain and decreased muscle strength [5–7]. Vitamin D deficiency and insufficiency is a prevalent worldwide problem [8]. Especially in northerly countries such as Denmark in which the absence of vitamin D from UVB sources during winter increases the risk of deficiency and insufficiency [9, 10]. Immigrants (first generation and descendants) living in Denmark as well as other Nordic countries are considered at risk of vitamin D deficiency due to factors such as skin colour, cultural clothing and dietary habits [11–13].

Bone tissue is a dynamic structure that undergoes constant remodelling, and optimal bone health is a balance between bone resorption and bone formation. In a case of chronic bone resorption, the bone mineral density (BMD) will decrease with time [14]. The bone turnover can be monitored by bone biomarkers in the blood, and is therefore a potential cost-effective method to complement BMD measurements in situations where BMD is not a feasible endpoint e.g. studies of shorter duration [14, 15].

Recent advances have located vitamin D receptors (VDR) within muscle tissue, supporting the assumption that vitamin D has an effect on muscle function [16, 17]. Some intervention studies have found an effect of vitamin D on muscle strength [18, 19] and some effect in the number of falls [20], while other studies were unable to see an effect [21]. These inconsistent trends may be related to the baseline serum 25(OH)D concentration and the effect of vitamin D supplementation is more pronounced on vitamin D deficient people (25(OH)D concentrations < 25–30 nmol/L) [18, 19]. Additionally, assessment of the effect of vitamin D on muscle strength has primarily been studied among elderly subjects (mainly community dwelling), and there is a lack of data on the effects of vitamin D on muscle strength in young adults deficient in vitamin D [22]. The mechanism of vitamin D and muscle function has not been agreed upon, however, it has been hypothesised that the active metabolite of vitamin D (1,25(OH)_2_D) vitamin D is involved in the regulation of the calcium flow within the muscle through genomic and non-genomic pathways and is thus important for the contraction of muscle fibres [5, 17].

We aimed to investigate the effect of 12 weeks of intervention with vitamin D fortified foods on markers of bone turnover and muscle strength in a population of women of Danish and Pakistani origin between 18 and 50 years of age, at risk of vitamin D deficiency. Our hypothesis was that 12 weeks of intervention with 30 μg vitamin D_3_/day would significantly improve muscle strength in a population of Danish and Pakistani women at risk of vitamin D deficiency.

## Methods

### Study design

This paper is based on intervention data from a food-based randomised controlled trial (RCT) *ODIN-FOOD*. The intervention was designed to assess the change in serum 25-hydroxyvitamin D_3_ as the primary endpoint following fortification, while measurements of muscle strength and bone turnover markers were secondary outcomes. The main study outcomes of the *ODIN FOOD* project have been included in a previous publication [23], we will refer to this publication throughout the methods for further details.

The present study was a three month double-blinded RCT. A total of 143 women of Danish (*n* = 71) and Pakistani (*n* = 72) origin between 18 and 50 years at risk of vitamin D deficiency were enrolled in the study (see Table 1). Details of the study design, recruitment (flow diagram), randomisation and blinding has been described previously [23]. The subjects were given four different vitamin D fortified foods contributing with 30 μg/day (intervention group) or non-fortified foods (placebo group). The intervention was performed during the winter months (Jan. - Apr.) to minimise interference from cutaneous produced vitamin D. Participants were seen before the intervention start (January 2016) and at the end of intervention after 3 months (April 2016). At the visits blood was drawn, anthropometrics, dietary vitamin D intake and muscle strength were measured. The assessed endpoints were hand grip strength, knee extension strength, number of chair-stands in a 30-s chair stand test, and concentrations of four different bone turnover markers (OC, BALP, P1NP, CTX).

**Table 1:** Baseline characteristics of participants by study group and ethnicity^1^

	Danish			Pakistani		
	Total (*n* = 66)	Placebo (*n* = 35)	Fortified (*n* = 31)	Total (*n* = 70)	Placebo (*n* = 37)	Fortified (*n* = 33)
Born in Denmark (%)	99	100	99	62	70	54
Years lived in Denmark^2^	–	–	–	20	21	18
Mean age in years (SD)	33 (11)	34 (11)	32 (11)	36 (9)	36 (9)	36 (10)
Age range (years)	18–50	18–50	18–50	18–50	18–50	18–47
Mean weight in kg (SD)	68 (13)	70 (15)	67 (11)	70 (12)	68 (13)	70 (12)
BMI (kg/m^2^)*	24 (5)	25 (5)	24 (4)	27 (5)	27 (5)	27 (5)
Mean serum 25(OH)D (nmol/L)	49.6 (18)	46.2 (19)	53.3 (17)	46.9 (22)	49.0 (23)	44.5 (21)
Mean plasma creatinine (μmol/l)	61.4 (10)	61.4 (9)	61.4 (10)	58.0 (9)	57.8 (10)	58.0 (8)
Median dietary vitamin D intake (μg/d)*	1.5 (1.0; 2.0)	1.5 (1.0; 2.0)	1.7 (1.0; 2.1)	1.1 (1.0; 2.0)	1.1 (0.8; 1.4)	1.0 (0.7; 1.7)
Median dietary calcium intake (mg/d)	465 (324; 688)	487 (309; 685)	442 (331; 692)	441 (260; 589)	422 (262; 547)	465 (259; 634)
Smokers (%)	14	14	13	4	3	6
Alcohol drinkers (%)^¶^	86	89	84	1.4	3	0
Wearing hijab (%)^¶^	0	0	0	44	43	45

^1^Means and SD unless otherwise specified. If non-normally distributed, medians and 25^th^ and 75^th^ percentiles.

^2^Years lived in Denmark on average, if not born in Denmark.

^*^Significant difference between Danish (all) and Pakistani (all) women in an unpaired t test (*P* < 0.05).

^¶^Significant difference between Danish (all) and Pakistani (all) women in a Pearson’s chi^2^ test.

Sample size was based on 90% power (alpha = 5%) to detect a change in 25(OH)D concentration of 20 nmol/L in the treatment group with a standard deviation (SD) of 23, and a drop-out rate of 20%.

### Participants

The participants were of Danish and Pakistani origin, recruited from the Copenhagen area, city and suburbs (Denmark, 55°N). The recruitment was carried out in the autumn of 2016 by e-mail, advertisements and networking, as described elsewhere [23]. Inclusion criteria were a low consumption of fish and fish products (less than weekly), a low intake of vitamin D-containing supplements (less than weekly), no use of tanning facilities, no planned sun-holiday (more southerly than 47°N) between October 2015 and May 2016. No upper limit of the concentration of vitamin D in the supplement was specified as long as the participants stopped taking them at the time of enrolment in the study. Exclusion criteria were pregnancy and breastfeeding, menopause, non-Danish speakers, serious diseases (cancer, server liver or kidney insufficiencies, sarcoidosis and other granulomatous diseases) and medicines affecting the vitamin D metabolism (steroids, antiepileptic, thyroid hormones, bisphosphonates, oestrogen).

### Study foods

The intervention foods were low-fat Milner cheese (gouda) and yoghurt (plain) produced and provided free of cost by FrieslandCampina in the Netherlands, eggs (Livskraft) produced and provided free of cost by Hedegaard in Denmark, and whole wheat biscuits produced by Smørum konditori (confectionary) with ingredients supplied free of cost by Lantmännen Cerealia, Denmark, with vitamin D_3_ supplied by DSM nutritional products, Switzerland. The study foods were either placebo or fortified with vitamin D_3_ with the aim of increasing the total vitamin D status in an effective and safe manner in both ethnic groups. Foods highly representative and acceptable in both traditional Danish and Pakistani kitchen were chosen. The daily portion sizes were 150 g yoghurt, 60 g cheese, 1 egg and 1 small crisp bread (9 g). The fortified and non-fortified foods have the same fat percentage and comparable content of nutrients, except for the content of vitamin D_3_.

Random samples from every batch were analysed for vitamin D content at the National Food Institute in Denmark and the total daily vitamin D content was 29 μg/day (further details on vitamin D content, metabolites and methods described elsewhere, [23]).

### Measurements

#### Blood procurement procedures

At each of the two study visits non-fasting blood samples were obtained between 9 am and 16 PM for the majority of the samples. Efforts were made to book the endpoint measurement at the same time as baseline visit for each participant.

#### Anthropometric measures and questionnaires

Anthropometric measures were height (cm) (wall mounted stadiometer, seca, Hamburg, Germany), weight (kg), waist-hip circumference (cm) (standard tape measure) and body composition (Tanita BC 418 MA, Tokyo, Japan), as described elsewhere [23]. At baseline the participants completed two questionnaires, a general background questionnaire containing questions regarding general health, physical activity (during work and leisure time, if the participants had a job) and lifestyle, and a semi-quantitative Food Frequency Questionnaire (FFQ) estimating the average daily intake of vitamin D and calcium. The FFQ was a retrospective questionnaire asking about the participants’ habitual dietary intake of vitamin D-rich foods over the 3 preceding months. The questionnaire contained the 8 food groups (fish, meat, milk and milk products, egg, cheese, bread, fats and pulses) contributing to the majority of dietary vitamin D (98%) and calcium (71%) found in a general diet [24]. The weekly compliance was monitored by a self-reported user-friendly compliance questionnaire as described in details elsewhere [23].

#### Markers of bone turnover

In this paper we used the bone markers OC, BALP and P1NP for the assessment of the bone formation and CTX as a marker of bone resorption, based on previous studies carried out in a similar population group [25, 26].

The biochemical analyses of the bone turnover markers OC, BALP, P1NP, and CTX were performed at the University Hospital Aarhus. OC had a precision of ±0.55 μg/L (SD) at a concentration of 19 μg/L and ± 2.75 μg/L (SD) at a concentration of 92 μg/L. BALP had a precision of ±0.45 μg/L (SD) at a concentration of 4.5 μg/L and ± 4.6 μg/L (SD) at a concentration of 45.9 μg/L. P1NP had a precision of ±1.1 μg/L (SD) at a concentration of 30 μg/L and ± 7.6 μg/L (SD) at a concentration of 205 μg/L. CTX had a precision of ±0.015 μg/L (SD) at a concentration of 0.26 μg/L and ± 0.03 μg/L (SD) at a concentration of 0.59 μg/L.

#### Muscle function

Both upper and lower limb muscle function were measured using different physical tests. Upper limb muscle strength was measured as isometric hand grip strength (measured in kg) using a digital hand dynamometer (SAEHAN Corporation, South Korea) which have previously been demonstrated to have high inter-rate and test-retest reliability [27]. Participants were seated on a chair with the arm in a 90° elbow flexion, and the forearm in mid-prone neutral position. The handle of the dynamometer was placed in position 2, thereby fitting the standard hand size for adults. All participants had two familiarisation trials prior to the maximal contraction test. A minimum of three maximal isometric contractions were conducted with the dominant hand with a 90-s break between each measurement. If the last contraction was the highest, the participants had up to two additional contractions. The instructor gently supported below the instrument to minimised an effect of gravity. Muscle strength was calculated as the mean of the three highest values obtained (averaged peak grip strength). The intra-assay coefficient of variation (CV) for hand grip strength was 4.0%.

Lower limb muscle strength was measured as isometric knee extension strength (measured in kg) using a digital handheld muscle tester (MicroFET2, Hoggan Scientific, USA). The MicroFET2 dynamometer was belt-stabilised to the table, so that the instructor would only have to concentrate on holding the dynamometer in the correct position on the ankle [28].The participants were seated on a table fitted with a non-skid material in an upright position with a 90° hip flexion and arms placed on the lap. The lower leg was hanging down about 1 cm from the edge of the table without touching the floor. The MicroFET2 dynamometer was placed on the front of the ankle, on the dominant leg, approximately 4 cm above the lateral malleoli. The participants were asked to stretch their leg as much as they could for approximately 5 s. The participants had two familiarisation trials where they were asked to stretch their leg using ≈ half of maximal power. This was followed by 3–5 maximal contractions lasting approximately 5 s with a 90-s break between each measurement. Muscle strength was calculated as the mean of the three highest values obtained. The intra-assay CV for knee extension strength was 4.2%.

Lower limb muscle function was also measured by a 30-s chair-stand test, as a measurement of anaerobic muscle function. Participants were seated on a chair (43 cm high, without arm rest). Arms were folded across the chest, and the number of full stands achieved in 30 s were recorded [29].

#### 25(OH)D-measurements and other biochemical measurements

Serum concentrations of total 25(OH)D (i.e., 25(OH)D_2_ plus 25(OH)D_3_) of all serum samples (baseline and endpoint visits) were measured at the Cork Centre for Vitamin D and Nutrition Research, University College Cork (UCC), using the ODIN core LC-MS/MS analytical platform for serum 25(OH)D, described in detail elsewhere [30]. The intra-assay CV of the method was < 5% for all 25(OH)D metabolites, whilst the inter-assay CV was < 6%. The LC-MS/MS method at UCC is certified by the Centre for Disease Control and Prevention’s Vitamin D Standardization Certification Program (VDSP).

Serum creatinine a marker of muscle mass [31] was measured at the University Hospital Aarhus. Creatinine had the following precisions at specific concentrations: At 81 μmol/L ± 7.4% (95% CI) and at 567.0 μmol/L ± 5.0% (95% CI) and biological variation of ±11.9% (95% CI).

### Statistical analysis

Results are shown as means (SD) unless otherwise specified. Descriptive statistics were calculated using a two-sample t test or the non-parametric Kruskal Wallis test for comparing the two ethnic groups. Categorical variables were compared using a Pearson’s chi-square test. Simple one-way ANOVA were used to test for significant baseline comparison across the four study groups (DK placebo, DK fortified, PA placebo, Pa fortified) and a subsequent Tukey HSD test showed which groups were responsible for the difference.

ANCOVA analysis of covariance was used to analyse the effect of the intervention on the respective outcome variables (muscle strength and markers of bone turnover). We adjusted for age, ethnicity, BMI and study group as well as the respective baseline variables. Finally, to check the model assumptions the standardized residuals of the models were assessed visually for normality, variance homogeneity and linearity. Compliance was estimated individually by dividing the individual amount of food with the individual self-reported compliance the final compliance is shown as the percentage consumed foods of delivered foods.

Statistical analyses were performed using RStudio for Windows [32] (Version 1.1.414 –© 2009–2018 Rstudio, Inc.) with a significance level of α = 0.05.

## Results

### Subject characteristics

Baseline characteristics relating to anthropometrics, dietary intake of vitamin D and calcium, vitamin D status are listed in Table 1, a flow diagram has previously been published [23]. The mean baseline vitamin D status among the women of Danish and Pakistani origin were 49.6 (18) and 46.9 (22) nmol/L, respectively (no ethnic difference *P* = 0.8). At baseline 9 and 24% of the participants of Danish and Pakistani origin, respectively, had a vitamin D status below 30 nmol/L (distribution data shown elsewhere [23]). The women of Pakistani origin had a slightly higher BMI and fat percentage compared to the women of Danish origin (t test, both *P* <  0.001, data not shown) and lower lean mass (t test, *P* < 0.001, data not shown).

Baseline self-reported physical activity and health are listed in Table 2. Among the participants of Danish origin 24% reported their health as “very good”, this was only the case with 7% among the participants of Pakistani origin. Accordingly, 33% the women of Pakistani origin reported “somewhat bad”, whereas only 4% of the women of Danish origin reported this. When asked to compare the overall health with others of the same age, 6 and 26% of the women of Danish and Pakistani origin, respectively, reported “worse”.

**Table 2:** Baseline self-reported physical activity and self-rated health

	Danish	Pakistani
*Total physical activity*		
Physical activity score, no job^1^, n (%)	n total DK = 3	n total Pa = 22
1: Sedentary	0 (0%)	3 (14%)
2: Light	1 (33%)	15 (68%)
3: Medium	2 (67%)	3 (14%)
4: Heavy	0 (0%)	1 (4%)
Physical activity score, job and leisure, n (%)	n total DK = 63	n total Pa = 48
1: Sedentary	5 (8%)	10 (21%)
2: Light	31 (49%)	28 (58%)
3: Medium	22 (35%)	9 (19%)
4: Heavy	5 (8%)	1 (2%)
Self-rated health, n (%)	n total DK = 66	n total Pa = 70
1: Very good	24 (36%)	5 (7%)
2: Somewhat good	37 (56%)	34 (49%)
3: Somewhat bad	4 (6%)	23 (33%)
4: Very bad	1 (2%)	8 (11%)
Self-rated health compared to others of same age, n (%)	n total DK = 66	n total Pa = 70
1: A lot better	6 (9%)	4 (6%)
2: Somewhat better	16 (24%)	21 (30%)
3: The same	40 (61%)	27 (38%)
4: Worse	4 (6%)	18 (26%)

^1^Participant divided according to job situation in order to determine physical activity during their daily activities. Participant with a job were asked about activity during work and leisure time, participants without a job were asked about activities throughout the day, the only difference were the formulation of the questions. Physical activity score is a mean of eight self-rated questions on physical activity and sedentary time. The questions on physical activity is adapted from a previous intervention study [33]

### Intervention effects on bone markers and muscle strength

Generally, the differences seen at baseline were also observed at endpoint. We saw no significant change in bone turnover markers following the intervention, unadjusted ANOVA (*P* > 0.05, Table 3). We saw no significant differences in the changes in muscle strength (Table 4), despite that the intervention was effective in increasing the mean serum 25(OH)D concentration from 53.3 (17) to 77.8 (14) nmol/L and from 44.5 (21) to 54.7 (18) nmol/L among the fortified Danish and Pakistani women, respectively (*P* < 0.05) (data shown in full in previous publication [23]).

**Table 3:** Markers of bone turnover: OC, CTX, BALP, P1NP at baseline, endpoint and the change following intervention^1^

	Danish		Pakistani		
	Placebo (*n* = 35)	Fortified (*n* = 31)	Placebo (*n* = 37)	Fortified (*n* = 33)	*P*^2^
OC (μg/L)					
Baseline	23.9 (9.6)^d^	25.2 (8.7)^bc^	19.6 (8.4)^c^	18.2 (5.6)^bd^	0.001
Endpoint	22.3 (9.0)	22.7 (6.8)^b^	19.5 (8.9)	17.4 (5.0)^b^	0.03
Change	−1.2 (4.2)	−2.3 (4.6)	−0.20 (4.0)	−0.5 (2.9)	0.19
CTX (ng/mL)					
Baseline	0.25 (0.14)	0.30 (0.15)	0.27 (0.19)	0.21 (0.1)	0.13
Endpoint	0.23 (0.14)	0.26 (0.12)	0.24 (0.16)	0.19 (0.1)	0.20
Change	−0.006 (0.1)	− 0.05 (0.1)	− 0.03 (0.1)	− 0.02 (0.1)	0.38
BALP (μg/L)					
Baseline	15.5 (7.0)	16.6 (5.2)	15.9 (5.2)	16.9 (7.9)	0.79
Endpoint	13.4 (5.4)	14.0 (4.4)	14.6 (4.9)	14.6 (6.0)	0.74
Change	−1.5 (3.5)	−1.5 (3.6)	− 1.4 (2.1)	− 1.8 (4.5)	0.97
P1NP (μg/L)					
Baseline	50.7 (25.2)	54.6 (19.6)	46.6 (19.6)	48.1 (17.2)	0.42
Endpoint	46.4 (23.8)	51.1 (16.8)	48.5 (21.0)	45.7 (14.7)	0.72
Change	−2.0 (7.9)	−2.6 (11.0)	1.7 (10.3)	−1.7 (10.4)	0.31

^1^Means and SD unless otherwise specified.

^2^*P* values for comparisons over the four study groups were determined with the use of a one-way ANOVA followed by Tukey’s HSD test. Significant comparisons a: DK Placebo vs DK Fortified, b: PA Fortified vs DK Fortified, c: PA Placebo vs DK Fortified, d: PA Fortified vs DK Placebo, e: PA Placebo vs DK Placebo, f: PA Placebo vs PA Fortified.

*OC* Osteocalcin, *BALP* Bone specific Alkaline Phosphatase, *P1NP* Procollagen type 1 amino-terminal propeptide, *CTX* C-terminal crosslinked telopeptide of type 1 collagen

**Table 4:** Muscle strength and body composition at baseline, endpoint and the change following intervention^*1*^

	Danish		Pakistani		
	Placebo (*n* = 35)	Fortified (*n* = 31)	Placebo (*n* = 37)	Fortified (*n* = 33)	*P*^2^
Handgrip strength (kg)					
Baseline	34.5 (4.2)^de^	33.2 (4.2)^c^	27.9 (6.6)^ce^	30.3 (6.2)^d^	< 0.001
Endpoint	34.07 (3.8)^de^	33.3 (4.3)^c^	28.0 (6.1)^ce^	30.3 (5.5)^d^	< 0.001
Change	−0.46 (2.3)	− 0.1 (3.0)	0.2 (3.0)	0.5 (3.5)	0.66
Knee extension strength (kg)					
Baseline	34.0 (5.7)^e^	36.5 (5.9)^bc^	27.7 (6.5)^ce^	31.1 (7.7)^b^	< 0.001
Endpoint	35.0 (5.9)^e^	37.5 (5.4)^bc^	27.5 (6.7)^ce^	31.2 (7.8)^b^	< 0.001
Change	0.3 (4.5)	1.0 (3.9)	−0.15 (5.0)	0.6 (4.3)	0.79
Chair-stand test (Mean rep/min)					
Baseline	23.4 (5.9)	24.0 (5.0)^c^	19.9 (5.5)^c^	22.6 (7.1)	0.04
Endpoint	24.7 (6.6)	26.8 (6.5)^c^	21.9 (5.9)^c^	23.9 (7.2)	0.045
Change	1.2 (3.0)	2.7 (3.6)	1.3 (3.4)	1.6 (3.6)	0.35
Mean BMI (kg/m^2^)					
Baseline	24.6 (5.1)	24.1 (4.0)^bc^	27.1 (24.8)^c^	27.4 (4.7)^b^	0.007
Endpoint	24.4 (4.3)	24.1 (3.9)^bc^	27.2 (5.1)^c^	27.2 (4.3)^b^	0.005
Change	0.1 (0.5)	0.2 (0.5)	0.2 (0.5)	0.1 (0.5)	0.97
Mean fat %					
Baseline	32.6 (7.6)^de^	30.1 (7.3)^bc^	37.1 (6.1)^ce^	37.6 (5.7)^bd^	< 0.001
Endpoint	32.0 (7.4)^de^	30.7 (7.1)^bc^	36.6 (6.4)^ce^	37.4 (5.6)^bd^	< 0.001
Change	−0.11 (1.9)	0.7 (2.3)	−0.3 (1.4)	0.16 (1.7)	0.20
Mean lean mass (kg)					
Baseline	46.5 (4.7)^e^	47.1 (3.0)^bc^	42.8 (4.7)^ce^	43.8 (4.7)^b^	< 0.001
Endpoint	46.5 (4.3)^de^	46.8 (3.0)^bc^	43.2 (4.8)^ce^	43.16 (4.1)^bd^	< 0.001
Change	0.22 (1.3)	−0.05 (1.3)	0.43 (1.0)	0.06 (0.87)	0.37

^1^Means and SD unless otherwise specified.

^2^*P* values for comparisons over the four study group were determined with the use of a one-way ANOVA followed by Tukey’s test. Significant comparisons a: DK Placebo vs DK Fortified, b: PA Fortified vs DK Fortified, c: PA Placebo vs DK Fortified, d: PA Fortified vs DK Placebo, e: PA Placebo vs DK Placebo, f: PA Placebo vs PA Fortified.

#### Linear models

The intervention with vitamin D fortified foods showed no effect on the markers of bone turnover OC, CTX, BALP and P1NP, analysed in an ANCOVA including the baseline covariates age, ethnicity, intervention group and BMI. Only the baseline concentration of each marker of bone turnover had a significant influence on the endpoint concentration of the specific bone marker (Table 5).

**Table 5:** The effect of intervention with vitamin D fortified foods on the markers of bone turnover OC, BALP, P1NP and CTX, analysed in an ANCOVA including the baseline covariates that may influence the outcome (age, ethnicity, intervention group and BMI)

Coefficients	Estimate	95% CI	*P*	Estimate	95% CI	*P*
Δ OC	Model 1 (minimal)			Model 2 (maximal)		
Intercept^1^	3.14	(0.95; 5.32)	0.005 (**)	6.53	(1.69; 11.4)	0.01 (*)
Baseline OC	−0.20	(− 0.27; − 0.12)	< 0.0001 (***)	− 0.21	(− 0.29; − 0.13)	< 0.0001 (***)
Ethnicity:						
Danish	–	–	–	–	–	–
Pakistani	0.50	(−0.87; 1.83)	0.50	−0.70	(− 0.67; 2.07)	0.31
Intervention group:						
Fortified	−0.57	(−1.87; 0.72)	0.39	−0.60	(−1.86; 0.66)	0.35
Placebo	–	–	–	–	–	–
BMI				−0.10	(− 0.25; 0.05)	0.20
Age				− 0.01	(−0.08; 0.06)	0.69
Δ BALP	Model 1 (minimal)			Model 2 (maximal)		
Intercept^1^	3.4	(1.93; 4.94)	< 0.0001 (***)	0.50	(−2.6; 3.59)	0.75
Baseline BALP	−0.32	(−0.40; − 0.24)	< 0.0001 (***)	−0.25	(− 0.33; − 0.17)	< 0.0001 (***)
Ethnicity:						
Danish	–	–	–	–	–	–
Pakistani	0.2	(−0.84; 1.23)	0.71	0.07	(− 0.93; 1.08)	0.88
Intervention group:						
Fortified	−0.03	(−1.06; 1.00)	0.95	0.19	(−0.76; 1.13)	0.70
Placebo	–	–	–	–	–	–
BMI				0.05	(−0.07; 0.16)	0.43
Age				0.02	(−0.03; 0.07)	0.42
Δ P1NP	Model 1 (minimal)			Model 2 (maximal)		
Intercept^1^	6.81	(1.78; 11.84)	0.005 (**)	13.4	(1.10; 25.8)	0.03 (*)
Baseline P1NP	−0.17	(− 0.25; − 0.09)	< 0.0001 (***)	− 0.19	(− 0.28; − 0.11)	< 0.0001 (***)
Ethnicity:						
Danish	–	–	–	–	–	–
Pakistani	1.88	(−1.52; 5.28)	0.28	1.79	(−1.78; 5.35)	0.32
Intervention group:						
Fortified	−1.36	(−4.75; 2.04)	0.43	−1.63	(−5.02; 1.76)	0.34
Placebo	–	–	–	–	–	–
BMI				0.04	(−0.37; 0.45)	0.85
Age				−0.18	(−0.36; 0.01)	0.07
Δ CTX	Model 1 (minimal)			Model 2 (maximal)		
Intercept^1^	0.06	(0.03; 0.10)	< 0.0001 (***)	7.2^−2^	(−0.03; 0.17)	0.15
Baseline CTX	−0.33	(−0.42; − 0.23)	< 0.0001 (***)	−0.33	(− 0.43; − 0.23)	< 0.0001 (***)
Ethnicity:						
Danish	–	–	–	–	–	–
Pakistani	−0.004	(−0.03; 0.02)	0.80	−0.003	(− 0.03; 0.03)	0.82
Intervention group:						
Fortified	−0.01	(−0.04; 0.02)	0.40	−0.01	(− 0.04; 0.01)	0.37
Placebo	–	–	–	–	–	–
BMI				−2.1^−4^	(−0.004; 0.003)	0.91
Age				−2.8^−6^	(−0.002; 0.002)	1.00

^1^The reference group is included in the intercept: Danish ethnicity and placebo study group

Considering the muscle strength tests, fortification with vitamin D did not have a significant effect on the muscle strength measured as hand grip strength, only the baseline hand grip strength had a significant influence on the endpoint hand grip strength (Table 6).

**Table 6:** The change in muscle strength measured as handgrip strength and knee extension strength and a 30 s chair-standing test, analysed in an ANCOVA including the baseline covariates that may influence the outcome (age, ethnicity, intervention group and BMI)

Coefficients	Estimate	95% CI	*P*	Estimate	95% CI	*P*
Δ Handgrip strength	Model 1 (minimal)			Model 2 (maximal)		
Intercept^1^	6.80	(3.56; 10.0)	< 0.0001 (***)	6.56	(2.32; 10.8)	< 0.003 (**)
Baseline handgrip strength	−0.21	(−0.31; − 0.12)	< 0.0001 (***)	− 0.22	(− 0.31; − 0.12)	< 0.0001 (***)
Ethnicity:						
Danish	–	–	–	–	–	–
Pakistani	− 0.53	(−1.63; 0.58)	0.35	− 0.63	(−1.82; 0.56)	0.30
Intervention group:						
Fortified	0.38	(−0.62; 1.37)	0.46	0.48	(−0.54; 1.50)	0.35
Placebo	–	–	–	–	–	–
BMI				0.01	(−0.11; 0.13)	0.88
Age				−0.003	(−0.05; 0.06)	0.99
Δ Knee extension strength	Model 1 (minimal)			Model 2 (maximal)		
Intercept^1^	8.66	(4.16; 13.16)	< 0.0001 (***)	9.93	(3.82; 16.21)	< 0.0001 (***)
Baseline knee extension strength	−0.24	(−0.37; − 0.12)	< 0.0001 (***)	− 0.25	(− 0.38; − 0.12)	< 0.0001 (***)
Ethnicity:						
Danish	–	–	–	–	–	–
Pakistani	−1.96	(−3.69; − 0.23)	0.03 (*)	−1.95	(−4.24; 0.54)	0.03 (*)
Intervention group:						
Fortified	1.23	(−0.34; 2.81)	0.12	1.21	(−0.94; 3.52)	0.13
Placebo	–	–	–	–	–	–
BMI				−0.02	(−0.20; 0.18)	0.87
Age				−0.03	(−0.11; 0.06)	0.46
Δ Chair-standing test	Model 1 (minimal)			Model 2 (maximal)		
Intercept^1^	2.54	(−0.22; 5.31)	0.07	5.90	(0.78; 11.0)	0.02 (*)
Baseline chair-standing	−0.05	(− 0.15; 0.06)	0.41	− 0.06	(− 0.17; 0.06)	0.30
Ethnicity:						
Danish	–	–	–	–	–	–
Pakistani	−0.56	(−1.86; 0.74)	0.39	−0.01	(−1.45; 1.24)	0.88
Intervention group:						
Fortified	0.94	(−0.34; 2.22)	0.15	0.93	(−0.34; 2.20)	0.15
Placebo	–	–	–	–	–	–
BMI				−0.16	(−0.31; − 0.004)	0.04 (*)
Age				0.02	(−0.05; 0.09)	0.56

^1^The reference group is included in the intercept: Danish ethnicity and placebo study group

## Discussion

Following the 12-week intervention with vitamin D fortification no significant changes in the concentration of the markers of bone turnover OC, BALP, P1NP and CTX. Additionally, no significant increase in muscle strength in the two fortified groups and no significant decrease in muscle strength of the placebo groups were measured.

We expected a low baseline vitamin D status among our study population (< 30 nmol/L) as a previous Danish study from 2008, also including women of Pakistani origin found a very low baseline vitamin D status (< 15 nmol/L) [11, 25], and because a recent Danish study found a large proportion of participants (*n* = 2565) with a serum 25(OH)D status below 50 nmol/L in at least one of the seasons [34], suggesting that insufficiency is prevalent. In order to recruit only women at risk of vitamin D deficiency in the present study, we used restrictive inclusion criteria allowing only women with a low intake of fish and supplements as well as a low UVB exposure to be enrolled in the study (described elsewhere in detail, [23]). Despite vitamin D exposure-specific inclusion criteria, the mean baseline vitamin D status of the participants in this trial were 49.6 (18) and 46.9 (22) nmol/L in participants of Danish and Pakistani origin, respectively. We saw that 9% of the participants of Danish origin and 24% of participants of Pakistani origin had a serum 25(OH)D concentration below 30 nmol/L at baseline (data shown in full length elsewhere [23]). In the previously mentioned study from 2008 by Andersen et al. the distribution of extremely deficient and deficient vitamin D status among the women of Pakistani origin were reported as serum 25(OH)D concentration ≤ 10 or > 10- ≤ 25 nmol/L, and the prevalence was 40 and 44%, respectively at baseline [11, 25]. This may indicate that the vitamin D status among women of Pakistani origin living in Denmark have improved during the last 10 years, although it is important to be aware of the present study may be affected by social selection bias as is the case with any other health- or nutrition-related intervention study.

Observational studies report increased bone resorption with vitamin D deficiency [35, 36]. Despite observations of increased bone turnover, RCT’s assessing the effect of vitamin D supplementation on markers of bone turnover have shown inconsistent results [25, 26, 37–39].

Our study differs from these previous RCT’s by including both women of Pakistani origin and Danish origin and we used four different vitamin D fortified foods instead of a vitamin D supplements. The relatively high baseline serum 25(OH)D concentration seen among the women of Danish and Pakistani origin of 49.6 (18) and 46.9 (22) nmol/L, respectively, may be a reason for not finding an effect of vitamin D supplementation on markers of bone turnover. When vitamin D status is above the suggested cut off optimal concentration (> 50 nmol/L), PTH concentration will be within the normal range and calcium will not be released from the bone matrix to support the calcium balance [36].

Observational data have shown correlations between vitamin D status and muscle strength [40], however, when studied in RCT’s the effects of vitamin D appear inconclusive [41]. The majority of the published studies have been performed in older adults (> 50 years). Two larger systematic review and meta-analyses including 17 and 30 RCT’s, respectively, found that the effect of vitamin D supplementation may only be present among vitamin D deficient people (Vitamin D concentrations < 25–30 nmol/L) [18, 19]. A smaller systematic review and meta-analysis assessing healthy young adults (18–40 y) included six RCT’s and one controlled study assessing upper and lower limb strength and vitamin D. The authors conclude that vitamin D supplementation had a significant positive effect on muscle strength, and also that the lack of studies in the young adult population calls for further studies [42].

A Danish study assessed the effects of high dose (2500 μg/week for 1 month followed by 2500 μg/month for 5 months) vitamin D supplementation in women of Arabic origin (*n* = 55) with a Danish control group (*n* = 22). The women of Arabic origin had a baseline serum 25(OH)D concentration of 6.7 (0.6) nmol/L compared to the women of Danish origin having a serum 25(OH)D concentration of 47.1 (4.6) nmol/L. A very low baseline knee extension strength (maximum voluntary) was found among the women of Arabic origin of 26.4 kg, which was 34% lower than the 32.7 kg among the women of Danish origin. After high dose treatment, the ethnic difference disappeared. In addition, a significant reduction in reports of muscle and bone pain among the women of Arabic origin [43].

In the present study the serum 25(OH)D concentration in the placebo groups decreased by 2.8 (9) nmol/L in the Danish placebo group and by 11.2 (12) nmol/L in the Pakistani placebo group, however we did not see a significant decrease in muscle strength and bone formation markers or increase of the bone resorption marker (CTX) following the intervention. Based on the above, the present study suggests that a vitamin D status of ≈ 50 nmol/L measured at winter time (start of January) would be sufficient to maintain healthy muscle and bone during the following winter months.

### Strengths and limitations

The ODIN FOOD study was carried out in winter which is a strength in relation to avoiding confounding from vitamin D originating from UVB sources as no cutaneous production of vitamin D occurs during the months of winter (October–April) at Danish latitudes [10, 44]. An additional strength was the real-life setting of the study and thereby the applicability of the results in a public health setting. A limitation affecting health and nutrition studies in general is the bias of social selection during recruitment, this may also be a factor in this study. The fact that the participants may be a selected group, must be taken into account when interpreting the results.

The study was designed to detect the effect of vitamin D fortification on serum 25(OH)D concentration and thus, the power calculation was made with vitamin D status as the response variable. This may affect the results of secondary endpoint analyses such as muscle strength and bone markers due to a too low power to detect relevant changes in the mentioned endpoints.

Several limitations are present when using markers of bone remodelling. Large pre-analytical and analytical variability are seen and markers of bone turnover are affected by modifiable factors such as circadian rhythm, season, ethnicity, fasting state, physical activity and menstrual cycle [14]. Additionally, there are no standardised assays for bone markers. Since we were not able to obtain fasting blood samples from the participants due to logistical reasons, we attempted to book the participant at the same hour of the day at the two different measurements in order to minimise the preanalytical variability.

With regard to the study duration, 12 weeks has been shown to be the minimum time used in previous studies and may in fact have been too short a period to detect any changes in muscle strength and bone markers [19, 26] especially if the participants are in a vitamin D replete state of vitamin D, little may change following an intervention with vitamin D [19].

At baseline, the participants would perform the muscle test for the first time, whereas at the endpoint measurement there may be some familiarity of the muscle tests. Despite performing familiarisation trials before the tests, and giving the participants up to two additional muscle tests trials if the last one were the highest we cannot rule out that this may influence the score because of inexperience and reluctance to exert full capacity of power at baseline, compared to the endpoint.

Although successfully developed for older adults [29], the 30 s chair-stand test has also been used on people within our target group [45]. However, this test may not be completely optimal for this target group because the participants may have memorised their own baseline test results and some may even have tried to improve their score at the endpoint test. Also, participants with good physical fitness performed the test so fast that some had problems with the balance as a result of this.

## Conclusion

Consumption of vitamin D fortified foods for 12 weeks did not result in significant changes of the bone turnover markers OC, BALP, P1NP and CTX. Muscle strength measured as hand grip strength, knee extension strength and chair-standing did not change significantly following the intervention.

## Data Availability

The data can be made available on reasonable request to the corresponding author.

## Abbreviations

25(OH)D: Serum 25-hydroxyvitamin D
ANCOVA: Analysis of co-variance
ANOVA: Analysis of variance
BALP: Bone specific Alkaline Phosphatase
BMD: Bone Mineral Density
CTX: C-terminal crosslinked telopeptide of type 1 collagen
DTU: Technical University of Denmark
FFQ: Food Frequency Questionnaire
IOF: International Osteoporosis Foundation
LC-MS/MS: Liquid chromatography-tandem mass spectrometry
OC: Osteocalcin
ODIN: Food-based solutions for optimal vitamin D nutrition and health through the life cycle
P1NP: Procollagen type 1 amino-terminal propeptide
RCT: Randomised Controlled trial
SD: Standard Deviation
UCC: University College Cork
VDR: Vitamin D receptor
VDSP: Vitamin D Standardization Program
